# Diagnostic Performance and Safety of Positron Emission Tomography Using ^18^F-Fluciclovine in Patients with Clinically Suspected High- or Low-grade Gliomas: A Multicenter Phase IIb Trial

**DOI:** 10.22038/aojnmb.2016.7869

**Published:** 2017

**Authors:** Toshihiko Wakabayashi, Toshihiko Iuchi, Naohiro Tsuyuguchi, Ryo Nishikawa, Yoshiki Arakawa, Takashi Sasayama, Keisuke Miyake, Tadashi Nariai, Yoshitaka Narita, Naoya Hashimoto, Osamu Okuda, Hiroshi Matsuda, Kazuo Kubota, Kimiteru Ito, Yoichi Nakazato, Kan Kubomura

**Affiliations:** 1 Department of Neurosurgery, Nagoya University, Graduate School of Medicine, Aichi, Japan; 2 Division of Neurological Surgery, Chiba Cancer Center, Chiba, Japan; 3 Department of Neurosurgery, Osaka City University Graduate School of Medicine, Osaka, Japan; 4 Department of Neuro-Oncology/Neurosurgery, Saitama International Medical Center, Saitama Medical University, Saitama, Japan; 5 Department of Neurosurgery, Kyoto University Graduate School of Medicine, Kyoto, Japan; 6 Department of Neurosurgery, Kobe University Graduate School of Medicine, Hyogo, Japan; 7 Department of Neurological Surgery, Faculty of Medicine, Kagawa University, Kagawa, Japan; 8 Department of Neurosurgery, Tokyo Medical and Dental University, Tokyo, Japan; 9 Department of Neurosurgery and Neuro-Oncology, National Cancer Center Hospital, Tokyo, Japan; 10 Department of Neurosurgery, Osaka University Graduate School of Medicine, Osaka, Japan; 11 Department of Neurosurgery, Juntendo Tokyo Koto Geriatric Medical Center, Tokyo, Japan; 12 Integrative Brain Imaging Center, National Center of Neurology and Psychiatry, Tokyo, Japan; 13 Division of Nuclear Medicine, Department of Radiology, National Center for Global Health and Medicine, Tokyo, Japan; 14 Department of Radiology, Tokyo Metropolitan Geriatric Hospital and Institute of Gerontology, Tokyo, Japan; 15 Department of Pathology, Hidaka Hospital, Gunma, Japan; 16 Clinical Development Department, Nihon Medi-Physics Co., Ltd., Tokyo, Japan

**Keywords:** Brain tumor, Clinical trial, ^18^F-fluciclovine, Glioma, Positron-Emission Tomography

## Abstract

**Objective(s):**

The study objective was to assess the diagnostic performance of positron emission tomography (PET) for gliomas using the novel tracer ^18^F-fluciclovine (anti-[^18^F]FACBC) and to evaluate the safety of this tracer in patients with clinically suspected gliomas.

**Methods:**

Anti-[^18^F]FACBC was administered to 40 patients with clinically suspected high- or low-grade gliomas, followed by PET imaging. T1-weighted, contrast-enhanced T1-weighted, and fluid-attenuated inversion recovery (or T2-weighted) magnetic resonance imaging (MRI) scans were obtained to plan for the tissue collection. Tissues were collected from either “areas visualized using anti-[^18^F]FACBC PET imaging but not using contrast-enhanced T1-weighted imaging” or “areas visualized using both anti-[^18^F]FACBC-PET imaging and contrast-enhanced T1-weighted imaging” and were histopathologically examined to assess the diagnostic accuracy of anti-[^18^F]FACBC-PET for gliomas.

**Results:**

The positive predictive value of anti-[^18^F]FACBC-PET imaging for glioma in areas visualized using anti-[^18^F]FACBC-PET imaging, but not visualized using contrast-enhanced T1-weighted images, was 100.0% (26/26), and the value in areas visualized using both contrast-enhanced T1-weighted imaging and anti-[^18^F]FACBC-PET imaging was 87.5% (7/8). Twelve adverse events occurred in 7 (17.5%) of the 40 patients who received anti-[^18^F]FACBC. Five events in five patients were considered to be adverse drug reactions; however, none of the events were serious, and all except one resolved spontaneously without treatment.

**Conclusion:**

This Phase IIb trial showed that anti-[^18^F]FACBC-PET imaging was effective for the detection of gliomas in areas not visualized using contrast-enhanced T1-weighted MRI and the tracer was well tolerated.

## Introduction

Gliomas are among the most prevalent primary brain tumors, occurring in approximately one in every four patients with primary brain tumors (1). According to the 2014 Report of the Brain Tumor Registry of Japan prepared by the Japan Neurosurgical Society, the estimated 5-year survival rate of patients with all grades of glioma is 49.6%, and patients with grade IV gliomas (Glioblastomas) have an extremely poor prognosis, with a 5-year survival rate of 10.1%, even after treatment with surgery and postoperative chemoradiotherapy (2).

Resection is the first treatment option for newly diagnosed gliomas (3), and the more completely the tumor is resected, the better the prognosis of the patient (4, 5). Therefore, an accurate evaluation of the tumor extent before or during surgery is essential to resect the glioma as thoroughly as possible, thereby improving treatment outcomes and prognosis (6, 7).

Although contrast-enhanced T1-weighted MRI is widely used for the diagnosis of gliomas in clinical practice, this technique only visualizes areas with a disrupted blood-brain barrier (BBB), not the tumor tissue *per se*. Furthermore, the BBB is not always entirely disrupted at the site of gliomas, because glioma tumor cells spread invasively; it has even been suggested that tumor cells may be present at points ≥ 3 cm outside the contrast-enhanced areas in high-grade gliomas, such as glioblastoma (8, 9). In addition, most low-grade gliomas cannot be visualized using contrast-enhanced T1-weighted imaging, and it is sometimes impossible to identify the location of the tumor accurately (10). Contrast-enhanced T1-weighted MRI does not have enough performance in the diagnosis of gliomas and more specific modalities for imaging gliomas are thus required.

Carbon-11-labeled methionine (^11^C-MET) is a positron emission tomography (PET) tracer that accumulates in tumor cells with increased amino acid metabolism, such as gliomas. ^11^C-MET passes through the BBB and visualizes the invasive extent of gliomas more accurately than regular contrast agents (11, 12). However, ^11^C-MET has an extremely short half-life (20 min) and can only be used in limited institutions equipped with a cyclotron for its production; therefore, it is not widely available for daily clinical practice (13).

^18^F-Fluciclovine (anti-[^18^F]FACBC, development code: NMK36) is a newly developed PET tracer that is a synthetic amino acid, 1-aminocyclobutanecarboxylic acid labeled with fluorine-18 (^18^F). The half-life of ^18^F is long enough (approximately 110 min) for delivery. Like ^11^C-MET, anti-[^18^F]FACBC accumulates in a manner that reflects amino acid metabolism in tissues (14, 15). Nonclinical and clinical studies have shown the tracer to have the following characteristics: 1) a high-degree of accumulation in gliomas after passing through the BBB, 2) a low-degree of accumulation in normal brain tissues, and 3) a low-degree of accumulation at sites of inflammation (16-19). A previous Phase IIa clinical trial in high-grade glioma patients showed that PET imaging using anti-[^18^F]FACBC can visualize gliomas outside the enhanced areas using contrast-enhanced T1-weighted MRI (20).

With this finding in the Phase IIa trial, we presumed that anti-[^18^F]FACBC-PET may be useful for the diagnosis of low-grade gliomas, which can be barely visible using contrast-enhanced T1-weighted MRI. We conducted a multicenter Phase IIb clinical trial to assess the diagnostic performance of anti-[^18^F]FACBC-PET in both high- and low-grade gliomas and to evaluate the safety of this tracer.

## Methods

Eleven medical institutions (listed in Supplementary Table 1) collaborated in this open-label study from October 2013 to July 2014. Conduct of the study was approved in advance by the Institutional Review Board at each institution. The study was performed in accordance with the ethical principles laid down in the Declaration of Helsinki and good clinical practice. Voluntary written consent for participation in the study was obtained from each subject prior to the start of the study. The study was registered as a clinical trial (No. JapicCTI-132289).

**Table 1:** Characteristic of the 40 subjects who were administered anti-[^18^F]FACBC

Item		Total
Number of subjects studied		40

Age (years)	Mean	55.0
	SD	15.8
	Median	58.0
	(Min/Max)	(21/84)
Sex	Male	31 (77.5)
	Female	9 (22.5)
Karnofsky performance status	100%	17 (42.5)
	90%	9 (22.5)
	80%	6 (15.0)
	70%	3 (7.5)
	60%	3 (7.5)
	50%	2 (5.0)
	≤40%	0 (0.0)
	Median	90%
pathological diagnosis at hospitals	Glioblastoma	15 (37.5%)
	Diffuse astrocytoma	8 (20.0%)
	Anaplastic astrocytoma	7 (17.5%)
	Oligodendroglioma	3 (7.5%)
	Oligoastrocytoma	2 (5.0%)
	Anaplastic ependymoma	1 (2.5%)
	Anaplastic oligodendroglioma	1 (2.5%)
	Tumor other than glioma	2 (5.0%)
	Resection not performed	1 (2.5%)

Numbers in parentheses are percentages.

### Patients

This study was conducted in patients who were aged 20 years or over and were suspected of having a high- or low-grade glioma based on their clinical symptoms, disease course, and MRI findings (T1-weighted, contrast-enhanced T1-weighted, and fluid-attenuated inversion recovery [FLAIR] [or T2-weighted] images) and who were scheduled to undergo brain tumor resection.

The following patients were excluded from the study: 1) patients who had received or were receiving treatment for the glioma (resection, chemotherapy, or radiotherapy); 2) patients who had received chemotherapy for malignant disease within the previous 5 years; 3) women who were pregnant, breast feeding, or possibly pregnant; 4) patients with hepatic or renal dysfunction; 5) patients with a Karnofsky performance status of ≤40; 6) patients with a history of drug hypersensitivity; 7) patients who had received anti-[^18^F]FACBC before participating in this study or who had received another investigational drug within 180 days of the acquisition of consent; and 8) patients who were judged by the investigators as being ineligible for participation in this study.

### Investigational drug

Anti-[^18^F]FACBC was manufactured in compliance with Good Manufacturing Practices for investigational new drugs and was supplied by Nihon Medi-Physics Co., Ltd. (Tokyo, Japan) according to a previously reported method (21) and was supplied at a dose of 185 MBq/2 mL (at assay time).

### Anti-[^18^F]FACBC-PET

The PET/CT system at each site is listed in Supplementary Table 1. Before the start of the study, the PET camera and peripherals at each site were checked using a phantom and were confirmed to meet the criteria established by the Japanese Society of Nuclear Medicine (22).

Patients fasted for at least 4 h and then were treated with 2 mL of anti-[^18^F]FACBC (actual dose of radioactivity: 186.1 ± 67.0 MBq, 106.0–287.1 MBq) using an intravenous injection, followed by a flush with physiological saline. Three-dimensional (3D) PET imaging of the head was performed for 10 min in 1 bed position using a PET camera starting between 10 and 20 min (13.1 ± 3.1 min) after the administration of anti-[^18^F]FACBC. Head CT imaging (tube voltage: 120 or 130 kV) was also performed for attenuation correction and/or image fusion immediately before or immediately after PET imaging. In some patients, attenuation correction was performed using an external radiation source.

### MRI for navigation

After anti-[^18^F]FACBC-PET imaging, T1-weighted (slice thickness: ≤2 mm), contrast-enhanced T1-weighted (slice thickness: ≤2 mm), and FLAIR (or T2-weighted) (slice thickness: ≤5 mm) images were obtained to plan the tissue collection locations for the resection. Supplementary Table 1 shows the MRI systems used in this trial. Cameras with a magnetic field strength of ≥1.5 T were used for imaging, and the T1-weighted and contrast-enhanced T1-weighted imaging sequences were performed using a 3D or 2D gapless mode under the same conditions.

### Tissue collection using a neuronavigation system

At the time of resection, three types of MR images and anti-[^18^F]FACBC-PET images were referred to using a neuronavigation system (iPlan Cranial [Brainlab AG, Feldkirchen, Germany] or StealthStation [Medtronic plc, Dublin, Ireland]), and a tissue specimen was collected from an area visualized as positive on anti-[^18^F]FACBC PET imaging but negative on contrast-enhanced T1-weighted imaging (hereinafter, Gd(-) PET(+) area) or in cases where a tissue sample was not available from a Gd(-) PET (+) area, from an area visualized as positive on both anti-[^18^F]FACBC-PET imaging and contrast-enhanced T1-weighted imaging (hereinafter, Gd(+) PET(+) area). These tissues were planned to be collected within the high-intensity areas on the FLAIR (or T2-weighted) images or the area to be resected. To minimize the influence of brain shift, tissue was collected using a needle biopsy from a small incision in the dura mater before resection.

When available, tissue samples were also collected using an open biopsy from areas that the investigator of each institute determined to be not visualized using either contrast-enhanced T1-weighted imaging or anti-[^18^F]FACBC-PET imaging (hereinafter, Gd(-) PET(-) areas) but were on route to the tumor and were clearly separate from Gd(+) and PET(+) areas.

Tissues were collected within 14 days after anti-[^18^F]FACBC-PET imaging for the patients with suspected high-grade gliomas and within 28 days for the patients with suspected low-grade gliomas.

### Pathological examination of the collected tissue specimens

Collected tissues were stained with hematoxylin-eosin and immunohistochemically for glial fibrillary acidic protein (GFAP), Olig-2, neurofilaments protein (NFP)-MH, nestin, Ki-67 (MIB-1), and mutant isocitrate dehydrogenase 1 protein (IDH1 R132H). A neuropathologist independent of the trial sites assessed whether the tissues were tumorous, and the tumors were diagnosed histologically according to the WHO classification (hereafter referred to as the central pathological diagnosis) (23). Other relevant findings such as cellularity measured using Gunmetry software (24), the Ki-67 [MIB-1] labeling index measured using Gunma-LI software (25), and the estimated proportion of tumor cells were evaluated.

In addition, a pathological diagnosis of the resected tissues according to the WHO classification was also made at each trial site (hereafter referred to as the definitive diagnosis).

### Identification of the tissue collection area

A radiologist independent of the trial sites who was an expert in the interpretation of MR images identified where the tissue was collected using contrast-enhanced T1-weighted images obtained at the time of tumor resection using the neuronavigation system and PMOD software (PMOD Technologies, Zürich, Switzerland).

Two radiologists independent of the trial sites, who were blinded to the backgrounds of the subjects and experts in the interpretation of images of nuclear medicine, assessed whether the tissues were collected from a high-signal intensity area on the FLAIR (or T2-weighted) images and from which area tissues were collected among the Gd(-) PET(+) area, the Gd(+) PET(+) area, or none of these areas on a consensus basis (hereafter referred to as the central imaging assessment).

### Assessment of safety

Before and 2-7 days after the administration of anti-[^18^F]FACBC, the patient’s subjective symptoms, objective findings, and results for the following examinations were recorded: resting 12-lead electrocardiography, examination of vital signs (blood pressure and pulse rate), hematologic tests (red blood cell count, hemoglobin, hematocrit, white blood cell count, differential white blood cell count [neutrophils, eosinophils, lymphocytes, monocytes, and basophils], platelet count, prothrombin time, activated partial thromboplastin time, and serum fibrinogen levels), biochemical blood tests (albumin, alkaline phosphatase, aspartate aminotransferase [glutamate oxaloacetate transaminase], alanine aminotransferase [glutamate pyruvate transaminase], γ-glutamyl transpeptidase, lactic dehydrogenase, total bilir-ubin, urea nitrogen, creatinine, total creatinine phosphokinase, total protein, albumin-globulin ratio, triglyceride, total cholesterol, blood glucose, sodium, potassium, chlorine, calcium, and phosphorus), and urinalysis (protein, glucose, and occult blood). The results were compared before and after administration to assess the safety of anti-[^18^F]FACBC.

### Statistical analyses

The positive predictive value of anti-[^18^F]FACBC-PET imaging for the diagnosis of glioma in the Gd(-) PET(+) areas and its 95% confidence interval (CI) were calculated as the primary endpoint. To examine whether this positive predictive value of anti-[^18^F]FACBC-PET imaging was statistically higher than the minimum diagnostic threshold established for the calculation of the sample size (average diagnostic accuracy of contrast-enhanced MRI for gliomas, 70%), a test was performed assuming a population proportion of 0.7.

The positive predictive value of anti-[^18^F]FACBC-PET imaging for the diagnosis of glioma in the Gd(+) PET(+) areas and its 95% CI were calculated as the secondary endpoint. SAS version 9.3 (SAS Institute Japan, Tokyo, Japan) was used for all the statistical analyses. The one-sided significance level was set at 2.5%.

## Results

### Patient characteristics

**Figure 1:**
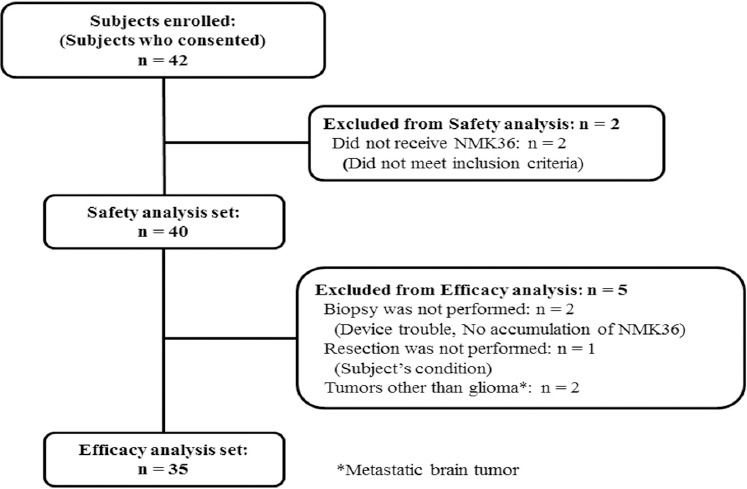
Patient characteristics. Forty-two subjects consented to participate in this study and were enrolled. Of these, 40 received anti-[^18^F]FACBC and were included in the safety analysis set. To assess the ability to visualize tumors using anti-[^18^F]FACBC-PET imaging, tissues were collected from 35 of the 40 patients who received anti-[^18^F]FACBC

Forty-two patients were enrolled from the 11 trial sites, and 40 were included in the safety analysis set (Figure 1). Two patient found not to meet inclusion criteria after consent did not receive anti-[^18^F]FACBC was excluded from analysis. The summarized patient characteristics of 40 patients who received NMK36 are shown in Table 1 and the details are shown in Supplementary Table 2.

**Table 2:** Histopathological diagnosis of the resected tissues

Subject	Age (years)	Sex	Central imaging assessment		Central pathological diagnosis	

			Gd	PET	Histological diagnosis	Grade
H01	59	Male	-	+	Anaplastic astrocytoma	3
			-	-	No evidence of tumor	-
H02	72	Male	+	+	Glioblastoma	4
			-	-	Infiltration of glioblastoma	-
H03	44	Male	+	+	Glioblastoma	4
H04	79	Female	+	+	Glioblastoma	4
H05	44	Male	-	+	Infiltration of glioma	-
			-	-	No evidence of tumor	-
H06	62	Male	+	+	Infiltration of astrocytoma	-
H07	34	Male	-	+	Infiltration of astrocytoma	-
H08	44	Male	-	+	Anaplastic oligoastrocytoma	3
H09	64	Male	-	+	Infiltration of astrocytoma	-
H10	70	Male	-	+	Severe infiltration of glioblastoma	-
			-	-	Infiltration of glioblastoma	-
H11	43	Male	-	+	Anaplastic astrocytoma	3
			-	-	Infiltration of astrocytoma	-
H12	55	Male	-	+	Infiltration of astrocytoma	-
H13	70	Male	-	+	Infiltration of glioma	-
H14	62	Female	+	+	Anaplastic oligodendroglioma	3
H15	49	Male	+	+	No evidence of tumor	-
H16	54	Male	+	+	Glioblastoma	4
H17	39	Female	-	+	Infiltration of glioma	-
L01	27	Male	-	+	Diffuse astrocytoma	2
			-	-	No evidence of tumor	-
L02	34	Male	-	+	Diffuse astrocytoma	2
			-	-	No evidence of tumor	-
L03	34	Male	-	+	Infiltration of low-grade glioma	-
			-	-	No evidence of tumor	-
L04	54	Male	-	+	Infiltration of astrocytoma	-
L05	62	Female	-	+	Oligoastrocytoma	2
L06	60	Female	-	+	Anaplastic oligodendroglioma	3
L07	48	Male	-	+	Oligodendroglioma	2
			-	-	Infiltration of oligodendroglioma	-
L08	67	Male	-	+	Diffuse astrocytoma	2
			-	-	No evidence of tumor	-
L09	80	Male	-	-	No evidence of tumor	-
L10	40	Female	+	+	Infiltration of diffuse astrocytoma	2
L11	70	Female	-	+	Anaplastic oligodendroglioma	3
L12	61	Male	-	+	Diffuse astrocytoma	2
L13	57	Male	-	+	Anaplastic oligodendroglioma	3
L14	21	Male	-	+	Oligodendroglioma	2
L15	68	Male	-	+	Anaplastic oligoastrocytoma	3
L16	62	Male	-	+	Anaplastic oligodendroglioma	3
L17	39	Male	-	+	Diffuse astrocytoma	2
L18	29	Male	-	+	Anaplastic astrocytoma	3
			-	-	No evidence of tumor	-

Tissues used to assess the diagnostic performance of anti-[^18^F]FACBC-PET for gliomas were collected from 35 of the 40 patients who received anti-[^18^F]FACBC; these 35 patients constituted the efficacy analysis set. Two patients in whom the biopsy was not performed, one patient who did not undergo resection, and two patients who had tumors other than gliomas were excluded from the efficacy analysis set. Tissues were collected from the Gd(-) PET(+) areas in 26 of the 35 subjects, and from the Gd(+) PET(+) areas in 8 subjects. In one subject from whom a tissue specimen was collected from a PET(+) area at the trial site, the area was determined as PET(-) at the central imaging assessment; therefore, this sample was excluded from the analysis of the positive predictive value of PET. In addition, tissues were collected from the Gd(-) PET(-) areas in 12 of the 35 subjects. Table 2 shows the results of the central imaging assessments and the results of the central pathological diagnoses of the tissues for the 35 patients.

### Diagnostic performance of anti-[^18^F]FACBC-PET

The positive predictive value of anti-[^18^F]FACBC-PET imaging for the diagnosis of glioma in the Gd(-) PET(+) areas was 100.0% (number of areas, 26/26; 95% CI, 86.8%–100.0%) (Table 3). The positive predictive value of anti-[^18^F]FACBC-PET imaging was tested and found to be significantly higher than the minimum diagnostic threshold (70%) established for calculation of the sample size (p < 0.001).

**Table 3:** Positive predictive value of anti-[^18^F]FACBC-PET imaging for the diagnosis of glioma

		Central imaging assessment	

		Gd(-), PET(+) area	Gd(+), PET(+) area
Central pathological diagnosis	Tumor	26	7
	Non-tumor	0	1
	Total	26	8

Positive predictive value of anti-[^18^F]FACBC-PET imaging (95% CI)		100.0% (86.8‒100.0)	87.5% (52.9‒97.8)

*One-sample binomial test: p < 0.001

**Figure 2:**
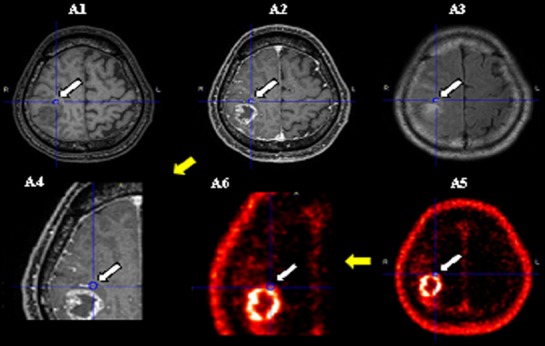
Case H12 (suspected high-grade glioma). T1-weighted (A1), contrast-enhanced T1-weighted (A2 and A4 [Close up of tumor]), FLAIR (A3), and anti-[^18^F]FACBC-PET (A5 and A6 [Close up of tumor]) images of a patient in whom tissue collected from a Gd(-) PET(+) area was histopathologically diagnosed as infiltration of astrocytoma. The Ki-67 index was 8.9% and approximately 10% of cells were tumor cells. (Arrow and circle of each image indicate tumor lesion and tissue sampling site.)

Figure 2 shows the MRI and anti-[^18^F]FACBC-PET images of a patient in whom tissue collected from a Gd(-) PET(+) area was histopathologically diagnosed as infiltration of astrocytoma.

The positive predictive value of anti-[^18^F]FACBC-PET imaging for the diagnosis of glioma in the Gd(+) PET(+) areas was 87.5% (7/8, 52.9%–97.8%) (Table 3). In addition, 8 (66.7%) of the 12 patients in whom tissues were collected from Gd(-) PET(-) areas were histopathologically confirmed as having no tumor, whereas the remaining 4 (33.3%) were diagnosed as having tumors. Of these 4 patients, the histopathological diagnoses and Ki-67 index of the tissues collected from the Gd(-) PET(-) areas and from areas visualized using anti-[^18^F]FACBC-PET (PET(+) areas) are shown in Table 4 for comparison. The histopathological diagnoses of tissues collected from PET(+) areas and from PET(-) areas were identical; however, the Ki-67 index tended to be higher in the tissues collected from the PET(+) areas.

**Table 4:** Comparison of tumors between PET(+) area and PET(-) area of 4 patients in whom tissues were collected from Gd(-) PET(-) areas and were histopathologically confirmed to be tumors based on a central pathological assessment

Subject	Pathological diagnosis of tissues collected from PET(+) areas		Pathological diagnosis of tissues collected from PET(-) areas	

	Histological diagnosis	Ki-67 (%)	Histological diagnosis	Ki-67 (%)
H02	Glioblastoma	42.6	Infiltration of glioblastoma	41.0
H10	Severe infiltration of glioblastoma	46.7	Infiltration of glioblastoma	34.5
H11	Anaplastic astrocytoma	15.7	Infiltration of astrocytoma	8.4
L07	Oligodendroglioma	9.7	Infiltration of oligodendroglioma	5.2

The Central pathological diagnoses for tissues collected from PET(+) areas in the same patients are shown for comparison.

**Figure 3:**
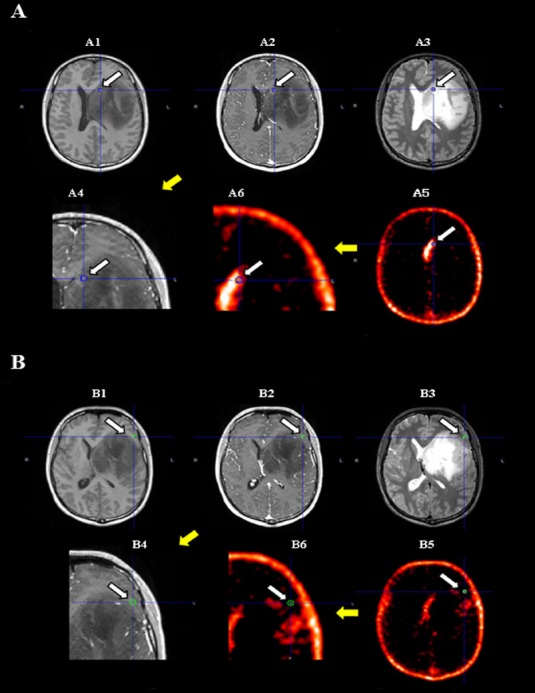
Case L18 (suspected low-grade glioma). T1-weighted (A1/B1), contrast-enhanced T1-weighted (A2/B2 and A4/B4 [Close up of tumor]), T2 (A3/B3), and anti-[^18^F]FACBC-PET (A5/B5 and A6/B6 [Close up of tumor]) images of a patient in whom (A) tissue collected from a Gd(-) PET(+) area was diagnosed histopathologically as anaplastic astrocytoma (Ki-67 index, 35.1%; approximately 80% of cells were tumor cells), and (B) tissue collected from a Gd(-) PET(-) area was histopathologically diagnosed as non-tumor (Ki-67 index, 0.0%; approximately 0% of cells were tumor cells). (Arrow and circle on each image indicate tumor lesion and tissue sampling site.)

Figure 3 shows the MR and anti-[^18^F]FACBC-PET images of a patient in whom tissue was collected from a Gd(-) PET(+) area and was histopathologically diagnosed as anaplastic astrocytoma. Tissue collected from a Gd(-) PET(-) area was histopathologically diagnosed as non-tumor.

**Figure 4:**
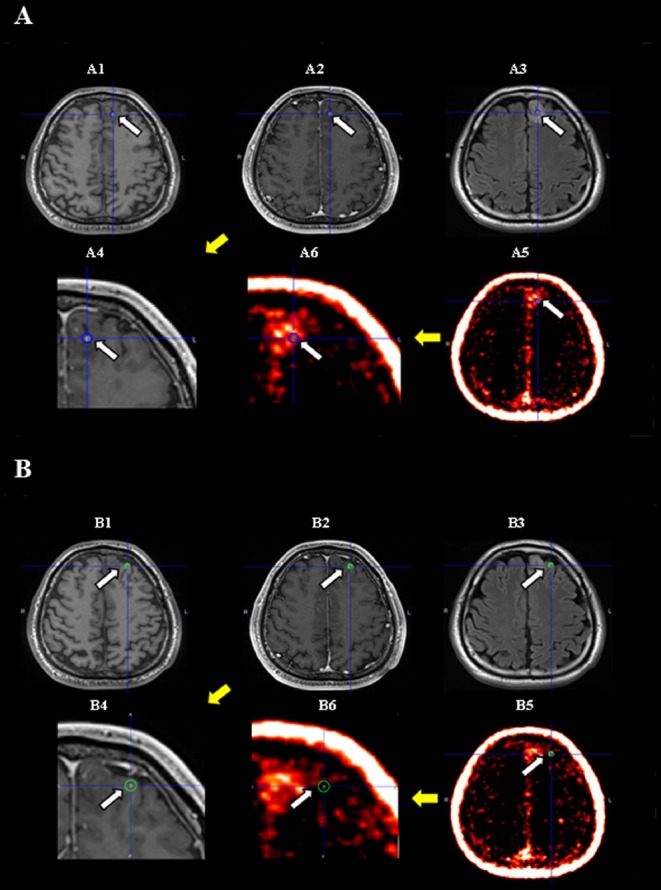
Case L07 (suspected low-grade glioma). T1-weighted (A1/B1), contrast-enhanced T1-weighted (A2/B2 and A4/B4 [Close up of tumor]), FLAIR (A3/B3), and anti-[^18^F]FACBC-PET (A5/B5 and A6/B6 [Close up of tumor]) images of a patient in whom (A) tissue collected from a Gd(-) PET(+) area was histopathologically diagnosed as oligodendroglioma (Ki-67 index, 9.7%; approximately 80% of cells were tumor cells) and (B) tissue collected from a Gd(-) PET(-) area was histopathologically diagnosed as infiltration of oligodendroglioma (Ki-67 index, 5.2%; approximately 5% of cells were tumor cells). (Arrow and circle of each image indicate tumor lesion and tissue sampling site.)

Figure 4 shows the MR and anti-[^18^F]FACBC-PET images of a patient in whom tissue was collected from a Gd(-) PET(+) area and was histopathologically diagnosed as oligodendroglioma. Tissue collected from a Gd(-) PET(-) area was histopathologically diagnosed as infiltration of oligodendroglioma.

### Safety of anti-[^18^F]FACBC

A total of 12 adverse events occurred in 7 of the 40 subjects (7/40, 17.5%) who received anti-[^18^F]FACBC. Each event occurred in a single patient (1/40, 2.5%), and none of the 12 events were serious.

Five events (ventricular extrasystoles, parosmia, glucose detected in urine, increased white blood cell count, and protein detected in urine) in 5 patients (5/40, 12.5%) were considered to be adverse drug reactions. These five events were mild and resolved spontaneously without medical treatment except for one case with glucose detected in the urine, the outcome of which remains unknown.

## Discussion

The positive predictive value of anti-[^18^F]FACBC-PET imaging in tissues collected from the Gd(-) PET(+) areas was 100%, indicating that anti-[^18^F]FACBC-PET allows the visualization of tumors that have invaded areas outside those visualized using contrast-enhanced T1-weighted MRI in patients with high-grade gliomas as well as the visualization of tumors not otherwise visualized using contrast-enhanced T1-weighted MRI in patients with low-grade gliomas. Therefore, anti-[^18^F]FACBC-PET imaging was capable of providing precise preoperative information regarding tumor extension.

The positive predictive value of a Gd(+) PET(+) area on anti-[^18^F]FACBC-PET imaging for glioma was as high as 87.5% (7/8) indicating that anti-[^18^F]FACBC-PET also allows visualization of tumors in areas visualized by contrast-enhanced T1-weighted imaging. In one patient, tissue collected from an area visualized on both anti-[^18^F]FACBC-PET and contrast-enhanced T1-weighted imaging was found to be non-tumorous. This may be attributable to the cystic nature of the lesion in question, with the needle sampling the outside of the cyst only. Accurate sampling is, of course, important for evaluating the performance of anti-[^18^F]FACBC-PET.

Histopathological diagnosis of the tissues collected from the Gd(-) PET(-) areas showed the absence of tumors in 8 of the 12 patients. For the four patients who were diagnosed as having tumors in the PET(-) area, the characteristics of the tissues collected from the PET(+) and PET(-) areas of each patient were compared histopathologically, revealing that the tissues collected from the PET(+) areas tended to have a more complete histological picture of the tumor and a higher Ki-67 labeling index than the tissues collected from the PET(-) areas (Table 4).

These results suggest that the areas visualized using anti-[^18^F]FACBC-PET should be aggressively resected during surgery even if the area was negative on Gd-MRI findings.

The usefulness of PET imaging using ^11^C-MET as the tracer for the diagnosis of gliomas has been reported. Surgical resection based on ^11^C-MET accumulation significantly improved the prognosis, as compared to resection based on MRI alone (4, 26), and persistence of ^11^C-MET accumulation after resection suggested a poor prognosis (11).

The efficacy of amino acid PET tracers labeled with ^18^F, such as O-(2-^18^F-fluoroethyl)-L-tyrosine (^18^F-FET) and 3,4-dihydroxy- 6-^18^F-fluoro-L-phenylalanine (^18^F-FDOPA) have also been reported for detecting primary and recurrent tumors, grading tumors, validating treatments, and predicting the prognosis (27, 28).

Although few studies have compared the performances among amino acid tracers, some overviews have suggested that they have the similar efficacy in the diagnosis of brain tumors (29, 30).

In the present trial, 12 adverse events occurred in 7 (17.5%) of the 40 patients who received anti-[^18^F]FACBC; of these, 5 events in 5 patients (12.5%) were considered to be adverse drug reactions. None of the events were serious. These findings suggest the good tolerability of anti-[^18^F]FACBC. In addition, no serious adverse drug reactions have been reported from four domestic and three foreign company-sponsored clinical trials involving anti-[^18^F]FACBC (31-34). Clinical studies other than company-sponsored trials have also been conducted, and no safety concerns have been reported (35).

## Conclusion

This Phase IIb trial showed that anti-[^18^F]FACBC-PET imaging was effective for the detection of gliomas in areas not visualized using contrast-enhanced T1-weighted MRI. Anti-[^18^F]FACBC was also well tolerated in patients with malignant glioma.

Since the complete resection of primary gliomas improves patient survival (2), it is very important to diagnose the tumor location and extent accurately before or during surgery (6, 7). The results of this study suggest that anti-[^18^F]FACBC-PET imaging may allow improved tumor resection completeness and may prolong patient survival by providing preoperative information supporting a more accurate estimate of the extent of tumor resection.
